# The application of eye-tracking in music research

**DOI:** 10.16910/jemr.11.2.1

**Published:** 2019-02-15

**Authors:** Lauren K. Fink, Elke B. Lange, Rudolf Groner

**Affiliations:** University of California, Davis Davis, CA, USA; Max Planck Institute for Empirical Aesthetics, Frankfurt, Germany; University of Bern, Switzerland

## Abstract

Though eye-tracking is typically a methodology applied in the visual research domain, recent studies suggest its relevance in the context of music research. There exists a communityof researchers interested in this kind of research from varied disciplinary backgrounds scattered across the globe. Therefore, in August 2017, an international conference was held at the Max Planck Institute for Empirical Aesthetics in Frankfurt, Germany,to bring this research community together. The conference was dedicated to the topic of music and eye-tracking, asking the question: what do eye movements, pupil dilation, and blinking activity tell us about musical processing? This special issue is constituted of top-scoring research from the conference and spans a range of music-related topics. From tracking the gaze of performers in musical trios to basic research on how eye movements are affected by background music, the contents of this special issue highlight a variety of experimental approaches and possible applications of eye-tracking in music research.

## Introduction

Music is an integral component of human society that serves a variety of emotional and social functions. Given its near ubiquitous role in cultures across the globe, music, when considered as an object of study, has the ability to provide insights into myriad biological and behavioral processes. Though music is typically conceived of as an auditory art form, the role of other senses, such as vision, in shaping musical experiences should not be ignored. We focus here on the application of eye-tracking in music research.

During recent years, there is a growing enthusiasm for applying eye-tracking methodology to study a range of musical processes. Besides measuring movements of the eyes (e.g., saccades, fixational eye-movements), eye-tracking captures two other types of movements: the muscles of the iris (pupil dilation) and of the eyelid (blinks). While eye movements are clearly relevant in tasks like music reading, it is less common that researchers have looked at the gaze, pupil, and blinks for insights into music perception, cognition, and engagement. On a basic level, studying vision in conjunction with audition allows the characterization of cross-modal interactions between these two sensory systems. Further, because different oculomotor movements are known to reflect the activity of specific neurotransmitter systems, e.g. blinks / dopamine, pupil size / norepinephrine, researchers can use eye-tracking to gain insights into the neural processes underlying specific musical activities. On an interpersonal level, the eyes are an important cue for social interaction and communication of emotion; one can therefore expect their role in musical social-emotional engagement to be similarly powerful.

In August 2017, the first conference on Music and Eye-Tracking was held at the Max Planck Institute for Empirical Aesthetics in Frankfurt, Germany. The conference brought together over 60 experts from psychology, neuroscience, and all fields of music research, with a common interest of investigating musical processing using eye-tracking or combining eye-tracking with other methods. This conference provided a platform for researchers to communicate with one another, to foster discussions about methods, and to continue refining models and theories of musical processing with insights from eye-tracking. For example, Dr. Bruno Laeng delivered the keynote address about using pupillometry to index autonomic nervous system activity and emotional / aesthetic evaluation of music. Information about the conference, including the program and abstracts, can be found at https://ae.mpg.de/met17. The conference sparked new, interdisciplinary ideas for collaboration and clearly highlighted the usefulness of eye-tracking methodologies in musical contexts. With this special issue, we hope to highlight some of the research presented at the conference and the potential of this young and growing research area.

## The Special Issue

Empirical data presented at the conference spanned a broad range of musical perspectives and experimental methodologies, from basic laboratory research on working memory and attention, to mobile eye-tracking in real-world musical contexts, such as during a music lesson or a musical performance. The content of this special issue is organized into four broad topics: music reading, music performance, music and visual processing, and pupillary responses to music. Each will be discussed in turn.

### Music reading

The topic of music reading opens the special issue, reflecting its initial role in bringing eye-tracking into the music research domain. Sight-reading – a special case of music reading that involves performing from a musical score at first sight – is the focus of the first two articles in this section. Puurtinen (1) provides a methodological review of sight-reading studies incorporating eye-tracking, from 1994 to 2017. She discusses critical discrepancies in the extant literature, namely, the choice of stimuli used in sight-reading studies, the protocol under which stimuli are sight-read, and the techniques employed in cleaning and statistically analyzing both the performance and the ocular data.

Huovinen, Ylitalo, and Puurtinen (2) introduce a novel dependent measure—the “Eye-Time Span”—which analyses ocular motor behavior relative to musical time, rather than to motor performance. They use the Eye-Time Span to investigate the effect of visual salience and musical complexity on eye-movement control.

Finally, in a study of cross-model integration during music reading, Drai-Zerbib and Baccino (3) investigate eye movements of experts vs. non-experts tasked with detecting deviations between read and heard melodies. In sum, the articles in this section outline experimental considerations, paradigms, and dependent measures that are very valuable for moving the field of music reading research forward, particularly by motivating increased harmonization of research standards.

### Music performance

The second group of papers in the special issue focuses on music performance in naturalistic contexts. In all three papers, mobile (head-mounted, glasses-based) eye-tracking is used to allow free-range motion of participants. The first paper in this section is methodological: Burger, Puupponen, and Jantunen (4) discuss issues involved in synchronizing mobile eye-tracking with motion capture and present a novel technique to align these two data series. They report results from a validation study that assesses the reliability of their synchronization technique.

The remaining papers in this section take an applied approach, using mobile-eye tracking to record ocular behavior of live music performances. Vandemoortele, Feyarts, Reybrouck, de Bièvre, Brône, and de Baets (5) explore the gaze behavior of four musical trios—each consisting of a clarinetist, violinist, and pianist—performing multiple iterations of the same musical fragment. The authors are particularly interested in the number of gazes between musicians as potential instances of social and/or musical coordination, and whether gaze patterns remain consistent across multiple rehearsals of the musical fragment.

In a cross-cultural comparison, Marandola (6) analyzes performances of Cameroonian percussionists vs. Canadian, conservatory-trained ones. He is interested in whether the eyes and hands are synchronized during xylophone performance in a consistent way, whether gaze behavior reflects anticipation of upcoming music to be played, and if performance speed affects gaze durations. Marandola discusses technical issues in recording mobile eye-tracking data and relating it to both the instrument keyboard and musical material, while also introducing novel dependent measures, like the “Eye-Stroke Span,” as a means to analyze gaze behavior in score-free conditions.

The research presented in this section is highly innovative and inspiring, on the one hand, and, on the other hand, it highlights methodological challenges in recording, synchronizing, and analyzing performers’ mobile eye-tracking data in conjunction with other data streams and/or musical events. These challenges, now clearly identified, must be addressed by the eye-tracking community in the future. In addition to the continued development of affordable, mobile eye-tracking devices with high measurement accuracy, the papers presented here will certainly motivate further research in this important field.

### Music and visual processing

The third section of the special issue centers around visual processing activities (reading, scanning, scene viewing, and watching movies) and how relevant or irrelevant musical stimuli affect them. Two of the papers focus on the effect of irrelevant background music on eye-movement control. Franěk, Šefara, Petružálek, Mlejnek, and van Noorden(7)compare eye movement behavior during scene viewing of urban or natural scenes, with a slow or fast pop song as background music. In their account, music affects eye movements because of its inherent ability to motivate physical activities. Lange, Pieczykolan, Trukenbrod, and Huestegge (8) investigate eye-movement control during reading or visual scanning, with the tasks accompanied by a simple, trance-like piece, presented at one of four different tempi. They suggest that an oculomotor saccade timer can be affected by irrelevant background music during highly automatized oculomotor processes, although effects are small.

Switching to the role of music in movie perception, Hammerschmidt and Wöllner (9) manipulated not only musical tempo but also playback speed of the visual scenes (slow versus real-time motion). They explore how music and playback speed affect eye movements, pupil dilation, and eyeblinks.

Leaving the research question of how music directly affects visual processing, the section shifts to another context of music and visual processing: viewing dance performance. Ponmanadiyil and Woolhouse (10) examine the eye movement behavior of novice vs. expert Bharatanatyam dancers viewing both narrative and non-narrative Bharatanatyam dance. They ask whether expertise is reflected in eye movement statistics and whether different styles of dance evoke different eye movement behavior.

Although the four papers in this section are diverse in their experimental designs, they all provide insight into the ways in which ocular motor behavior is affected by factors like musical tempo, expertise, and the visual task at hand.

### Pupillary responses to music

In the final section of the special issue, the focus is on pupillometry, i.e. the measurement of pupil size. Fink, Hurley, Geng, and Janata (11) use a linear oscillator model to predict the continuous pupillary response over time, as well as perceptual thresholds, while participants detected subtle changes within rhythmic musical patterns. They investigate entrainment of the pupil to auditory rhythm and also examine the pupil dilation response (PDR) as a marker of auditory deviance detection.

The PDR is likewise of interest to Liao, Yoneya, Kashino, and Furukawa(12), who analyze its relationship to surprising moments, as well as changes in loudness, during listening to classical, jazz, and rock music. In combination, these two papers underscore the ability of pupillometry to index various aspects of musical processing.

## Conclusion & Future Directions

Considered as a whole, this special issue on music and eye-tracking provides an initial but non-exhaustive overview of where the field stands in applying eye-tracking in music research. Additionally, these articles envision what might be possible in the future, uncovering challenges to confront along the way. Many authors assert the necessity of agreeing upon standardized experimental procedures and dependent measures to increase comparability within the music research community and beyond to other research domains. It is clear that, in a small niche like ours, communication and collaboration between researchers must be enhanced to move the field forward.

We hope that interest in the application of eye-tracking in music research will continue to grow, while simultaneously becoming methodologically refined. We are excited to follow new developments and look forward to hosting another Music and Eye-Tracking (MET) conference at the Max Planck Institute for Empirical Aesthetics in summer 2020.

### Acknowledgements

We wish to thank all of the authors of this special issue for their novel contributions, and especially the reviewers for their careful reading of the submissions and productive, thoughtful feedback.

### Ethics and Conflict of Interest

The author(s) declare(s) that the contents of the article are in agreement with the ethics described in http://biblio.unibe.ch/portale/elibrary/BOP/jemr/ethics.html and that there is no conflict of interest regarding the publication of this paper.
